# The complete plastome sequence of *Amorphophallus allenii* sheds light on intrageneric phylogeny of *Amorphophallus*

**DOI:** 10.1080/23802359.2026.2680786

**Published:** 2026-06-08

**Authors:** Haoliang Shi, Wei Wang, Ying Qi, Penghua Gao, Min Yang, Yongteng Zhao, Feiyan Huang, Jiani Liu, Jianrong Zhao, Lifang Li, Lei Yu

**Affiliations:** Yunnan Key Laboratory of Konjac Biology, College of Agronomy, Yunnan International Joint Laboratory of Konjac Resources Conservation and Utilization, Kunming University, Kunming, China

**Keywords:** Chloroplast genome, *Amorphophallus allenii*, Phylogenetic analysis

## Abstract

The chloroplast genome of *Amorphophallus allenii*, a rare species endemic to limestone habitats, was sequenced to resolve phylogenetic uncertainties within the genus. Using PacBio HiFi sequencing, we performed a de novo assembly and obtained a complete circular genome of 170,657 bp, featuring a typical quadripartite structure. The genome encodes 126 genes, including 81 protein-coding genes, 8 rRNA genes, and 37 tRNA genes. Maximum likelihood phylogenetic analyses based on whole plastome, coding sequence, and protein alignments robustly support the placement of *A. allenii* within the Continental Asia I clade, with *A. muelleri* identified as its closest relative. This study provides a reliable genomic resource that clarifies the species’ phylogenetic position and establishes a foundation for investigating evolutionary diversification and biogeographic patterns in the genus *Amorphophallus*.

## Introduction

The chloroplast is a semi-autonomous organelle essential for photosynthesis and plant metabolism (Ishida et al. 2014). Its genome is maternally inherited, structurally conserved, and evolves at a moderate rate. Compared to nuclear and mitochondrial genomes, chloroplast DNA exhibits clearer and more stable sequence variation, making it particularly suitable for phylogenetic reconstruction across broad and fine taxonomic scales (Wicke et al. 2011; Smith 2015).

**Figure 1.**
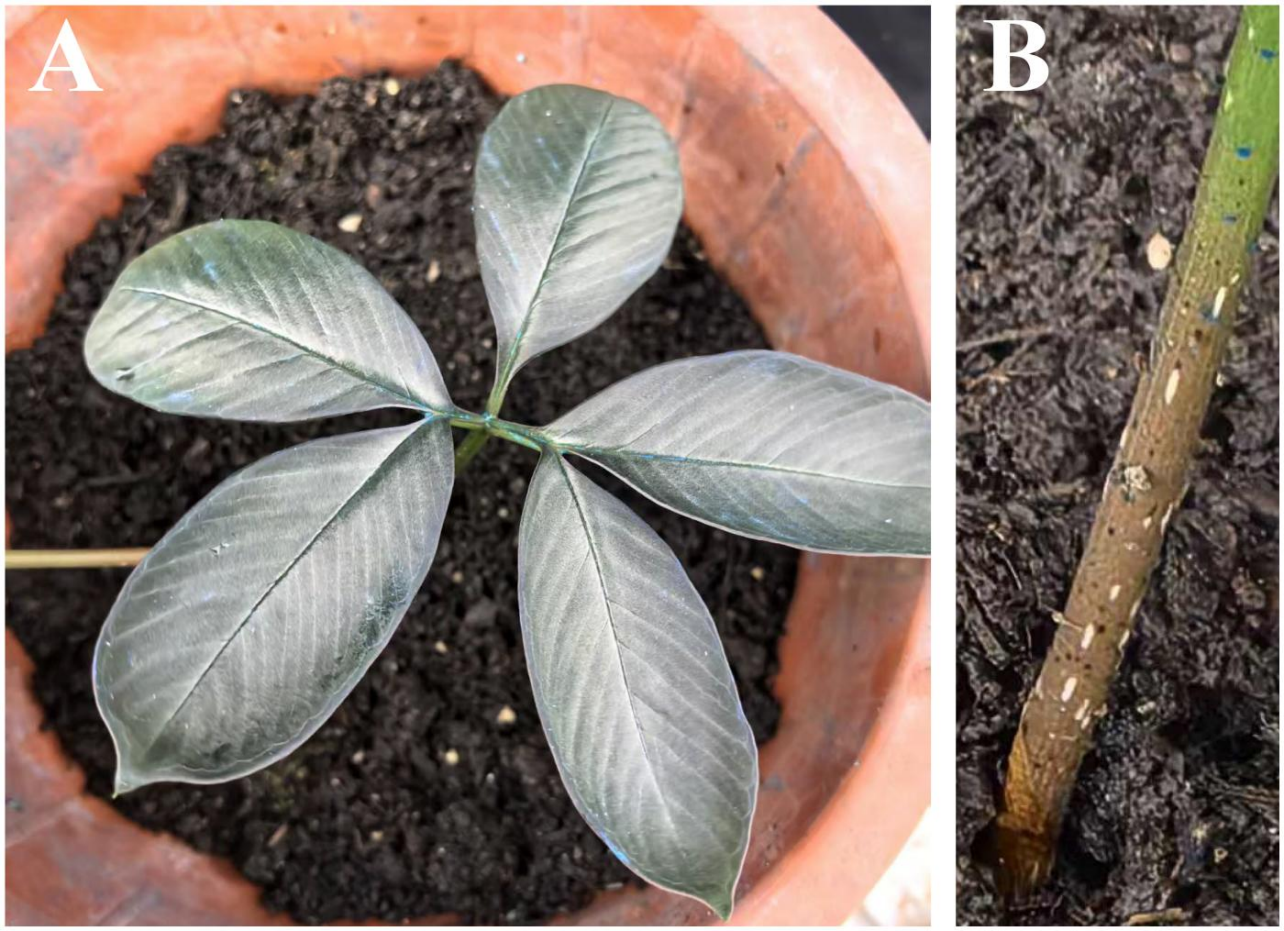
Plant morphology of *Amorphophallus allenii*. (A) The whole plant. The leaf surface is dark green with magenta margins, while the lower side is pale green to pale reddish-green. (B) Details of the petiole. The petiole has a smooth surface, exhibiting a dark olive-green to pale brown coloration, occasionally with a few creamy-white vertical lines. Photographed by Lei Yu, director of the Yunnan key laboratory of konjac biology.

*Amorphophallus*, a genus of perennial herbs in the family Araceae, comprises approximately 250 species primarily distributed across tropical and subtropical regions of Asia and Africa (Dong et al. 2024). These plants hold considerable economic value due to their corms, which are rich in glucomannan—a natural soluble dietary fiber shown to help regulate blood sugar, lower cholesterol, and support gut health. Additionally, many species are valued as ornamental plants in landscape design. *Amorphophallus allenii* A .Galloway, Malkm.-Huss., Prehsler & Claudel 2019 grows in fissures of limestone boulders and prefers dappled shade conditions (Galloway 2019). The petiole has a smooth surface, exhibiting a dark olive-green to pale brown coloration, occasionally with a few creamy-white vertical lines. The leaf surface is dark green with magenta margins, while the lower side is pale green to pale reddish-green (Figure 1) (Galloway 2019). Nevertheless, existing studies have been heavily biased toward a limited number of species from East Asia, notably southwestern China. There remains a significant lack of systematic investigation into the extensive species diversity present in other major centers, such as Africa and Southeast Asia. This geographical research gap substantially hinders a comprehensive understanding of the genus’s global evolutionary dynamics and biogeographical history.

This study reports the complete chloroplast genome of the rare species *A. allenii*. The generated high-resolution data serve as a key reference for resolving the complex phylogeny of the genus *Amorphophallus*, precisely placing *A. allenii* within its three major clades (African, Southeast Asian, and East Asian). Furthermore, by integrating its highly specialized habitat—limestone boulder crevices under dappled shade—genomic analysis helps elucidate potential genetic adaptations to nutrient-poor and low-light selective pressures.

## Materials and methods

### Plant material and DNA extraction

The plant material of *A. allenii* was cultivated at Kunming University. Fresh leaves were collected from the Konjac Genetic Resources Garden at the university’s Yunnan Province campus (24.97406° N, 102.79605° E). A specimen was deposited at the Herbarium of the Yunnan Urban Agricultural Engineering and Technological Research Center, Kunming University (contact person: Haoliang Shi; 1816148023@qq.com) under the voucher number YBMY20251020. Total genomic DNA was isolated from the fresh leaf tissue using a DNeasy Plant Mini Kit (Qiagen, Hilden, Germany) according to the manufacturer’s protocol.

### Library preparation, sequencing, and data processing

The purity and concentration of the extracted DNA were assessed using a NanoDrop^TM^ 1000 and a Qubit^TM^ 4.0 system (Thermo Fisher Scientific), respectively. A SMRTbell library with an insert size of approximately 15 kb was constructed using the SMRTbell Express Template Prep Kit 2.0 (Pacific Biosciences, CA, USA). The workflow included DNA shearing, purification with AMPure PB beads, removal of single-stranded DNA overhangs, DNA damage and end repair, hairpin adapter ligation, and final library purification. After quality control, the library was sequenced on the PacBio Revio platform (Pacific Biosciences). Raw subreads were processed into high-accuracy HiFi reads using the CCS algorithm (v6.0.0) with the following parameters: –minPasses 3 –minPredictedAccuracy 0.99 –maxLength 21000.

### Chloroplast genome assembly and annotation

HiFi reads (>3 Gb, average length >13 kb) were aligned to a reference chloroplast genome using minimap2 (v2.15-r905) to generate a PAF file (Li 2018). Reads with coverage below 50× were filtered out. The filtered reads were then assembled with Flye (v2.6) using the chloroplast genome of *Amorphophallus titanum* (NC_056329.1) as a reference, producing an assembly graph (Kolmogorov et al. 2020). The graph was visualized and edited in Bandage (v0.8.1) to remove redundant contigs and manually circularize the sequence, yielding the complete chloroplast genome of *A. allenii* (Wick et al. 2015). The genome was annotated with the Plastid Genome Annotator (PGA) followed by manual curation (Qu et al. 2019).

### Visualization

A circular map of the chloroplast genome was generated with OGDRAW (Greiner et al. 2019). Subsequently, using CPGView, we created a comprehensive circular map that integrates the genomic architecture along with cis- and trans-splicing gene information (Liu et al. 2023).

### Phylogenetic analysis

To determine the phylogenetic position of *A. allenii* within the genus *Amorphophallus*, we retrieved complete chloroplast genomes from NCBI for ten published congeneric species, two other Araceae species, and *Zea mays* as the outgroup. Phylogenetic analyses were conducted on three distinct datasets: (1) the complete chloroplast genomes, (2) aligned shared single-copy coding sequences (CDS), and (3) aligned shared single-copy proteins.

For the complete genome dataset, sequences were aligned with MAFFT v7.526 using the L-INS-i algorithm (–maxiterate 1000) (Katoh and Standley 2013), followed by trimming with trimAl under the automated heuristic mode (-automated1) (Capella-Gutiérrez et al. 2009). A maximum likelihood (ML) tree was then reconstructed from the processed alignment using IQ-TREE v1.6.10 under the TVM+F + R3 model (selected by ModelFinder), with 1000 bootstrap replicates (Nguyen et al. 2015). For the CDS and protein datasets, phylogenetic trees were automatically generated using the HiMT toolkit within TBtools-II, employing the GTR+F + I + G4 and cpREV models, respectively (Chen et al. 2023; Tang et al. 2025). All resulting phylogenetic trees were visualized and finalized for presentation using TBtools-II (Chen et al. 2023).

## Results

**Figure 2.**
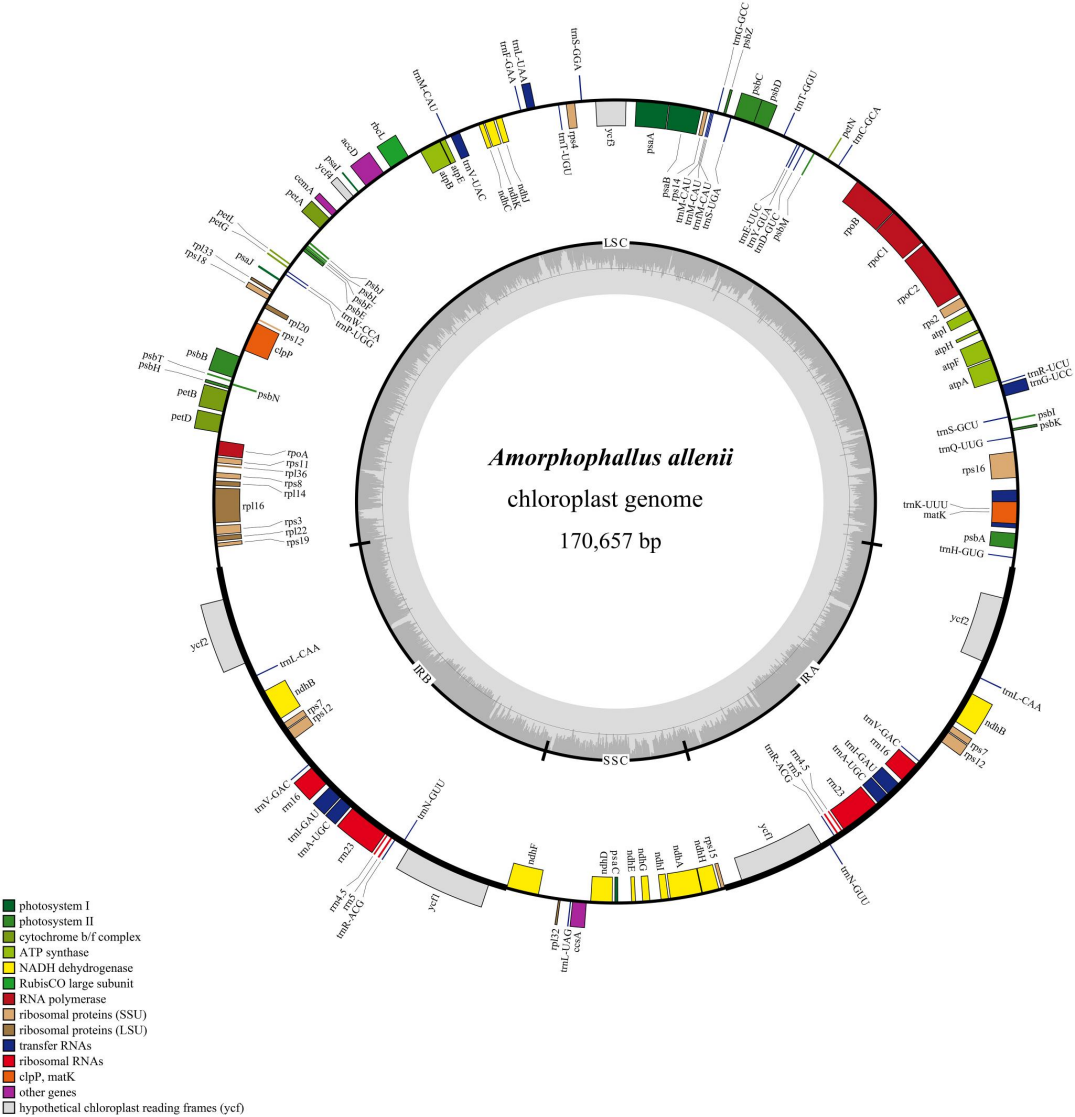
** ** A circular map depicting the *A. allenii* chloroplast genome. Genes are plotted in the outer circle according to their functional groups, and the quadripartite structure—including the chloroplast long-chain region (LSC), chloroplast short-chain region (SSC), and two intergenic regions (IR). Genes were color-coded according to their annotated function. The inner circle’s dark gray area represents the GC content of the chloroplast genome.

The assembled chloroplast genome exhibited an average coverage depth of 3,524.68×, with maximum and minimum depths of 7,999× and 1,999×, respectively (Figure S1). The complete chloroplast genome of *A. allenii* was 170,657 bp in length and displayed a typical quadripartite structure, consisting of a large single-copy (LSC) region (94,250 bp), a small single-copy (SSC) region (14,951 bp), and a pair of inverted repeat (IR) regions (IRa and IRb, each 30,728 bp). The overall GC content was 33.55% in the LSC, 29.90% in the SSC, and 38.44% in each IR region (Figure 2). A total of 126 genes were annotated, comprising 81 protein-coding genes (76 unique), 37 tRNA genes (29 unique), and 8 rRNA genes (4 unique).

**Figure 3.**
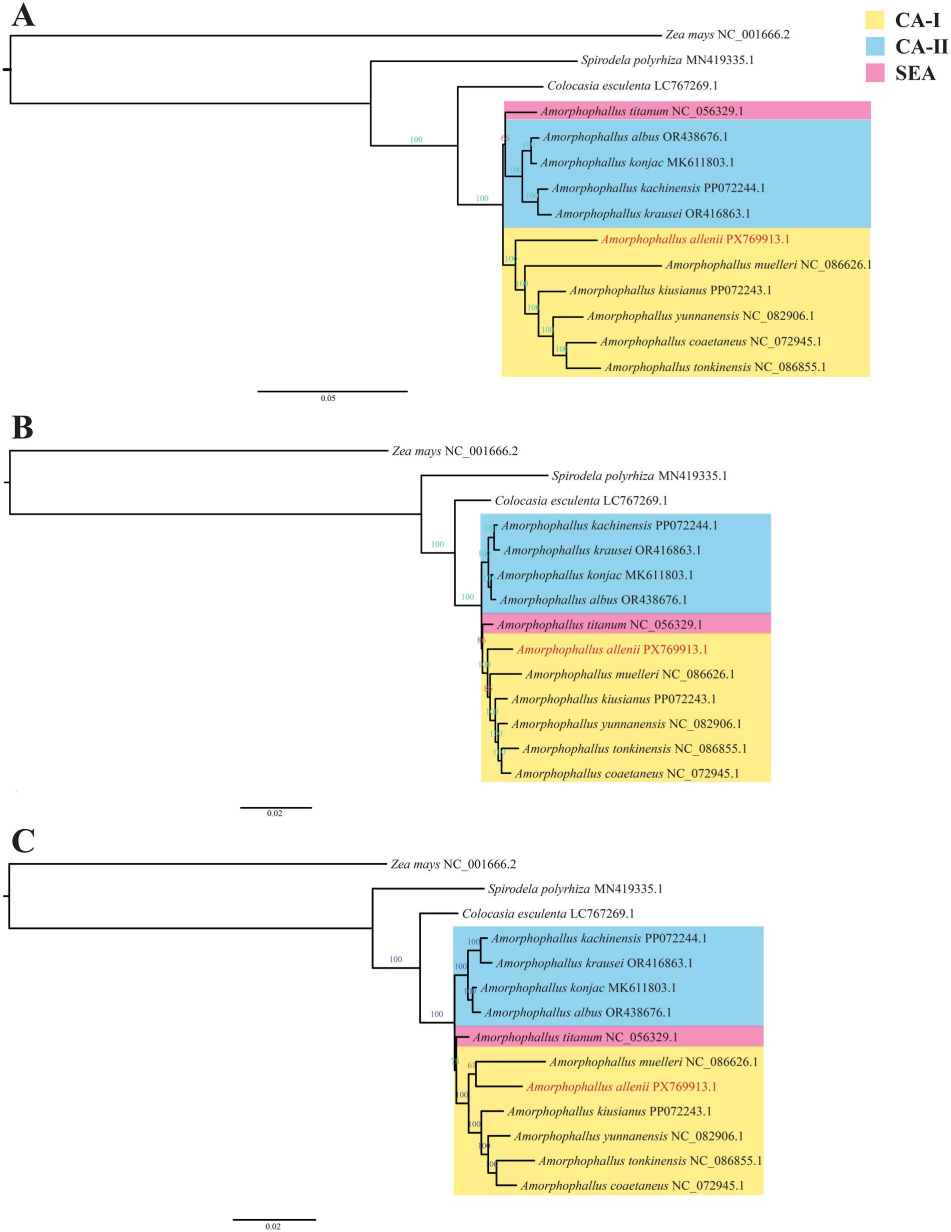
Phylogenetic trees of *A. allenii*. (A) Tree based on the complete chloroplast genome, (B) Tree based on shared single-copy CDS, (C) Tree based on shared protein sequences. *Zea mays* is used as an outgroup. The scale bar represents the number of substitutions per site. Numbers at each node represent the bootstrap values for 1000 replicates. *A*. *allenii* is marked in red. The chloroplast genomes used for tree construction are as follows: *A. allenii* PX769913.1 (this study), *A. konjac* MK611803.1 (Li et al. 2024a), *A. albus* OR438676.1 (Li et al. 2024b), *A. muelleri* NC_086626.1 (Li et al. 2024b), *A. krausei* OR416863.1 (Li et al. 2024b), *A. coaetaneus* NC_072945.1 (Gao et al. 2023), *A. titanum* NC_056329.1 (Liu et al. 2019), *A. yunnanensis* NC_082906.1 (Yin and Gao 2023a), *A. kachinensis* PP072244.1 (Gao and Yin 2024a), *A. kiusianus* PP072243.1 (Gao and Yin 2024b), *A. tonkinensis* NC_086855.1 (Yin et al. 2024), *Spirodela polyrhiza* MN419335.1 (Zhang et al. 2020), *Colocasia esculenta* LC767269.1 (Takaesu et al. 2025), *Z. mays* NC_001666.2 (Maier et al. 1995).

Among these, 14 genes contained a single intron, and two genes (*ycf3* and *clpP*) possessed two introns each (Figure S2). Additionally, the trans-splicing gene *rps12* was identified, whose three exons are distributed with two reside within the Inverted Repeated regions, forming a repetitive structural arrangement (Figure S3). Three maximum likelihood phylogenetic trees constructed from three datasets (complete chloroplast genomes, shared single-copy coding regions, and shared single-copy proteins) across 14 species indicate that the genus *Amorphophallus* is monophyletic. Furthermore, 11 species within *Amorphophallus* are further divided into 3 subclades in all three phylogenetic trees (CA-I, CA-II and SEA). Within this framework, *A. allenii* shares a subclade with *A. muelleri*, *A. kiusianus*, *A. yunnanensis*, *A. coaetaneus*, and *A. tonkinensis* (bootstrap = 100), indicating their close phylogenetic relationships. All three phylogenetic trees provide strong support for the classification system of the genus *Amorphophallus* (Figure 3).

## Discussion and conclusion

The complete plastome of *A. allenii*, yielding a 170,657 bp circular molecule with a canonical quadripartite structure (aligns with the established size range of 161,647-177,076 bp for the genus) is comprising a large single-copy (LSC) region of 94,250 bp, a small single-copy (SSC) region of 14,951 bp, and a pair of inverted repeat (IR) regions (IRa and IRb), each 30,728 bp in length. Comparative analysis across *Amorphophallus* species indicates that the LSC region of *A. allenii* is longer than that of its congeners—including *A. krausei* (91,983 bp), *A. albus* (93,177 bp), *A. konjac* (93,443 bp), *A. yunnanensis* (92,149 bp), *A. tonkinensis* (90,705 bp), *A. kachinensis* (92,030 bp), and *A. paeoniifolius* (93,951 bp). In contrast, its SSC region is shorter than that of the same set of species, which range from 15,013 bp (*A. paeoniifolius*) to 21,575 bp (*A. konjac*). This pattern shows an observable lengthening of the LSC concurrent with a shortening of the SSC in *A. allenii* relative to other examined species within the genus (Yin and Gao 2023a; 2023b; Gao and Yin 2024a; Li et al. 2024a; 2024b; 2024c). Although the repertoire and types of unique protein-coding genes in *A. allenii* are largely conserved relative to other species within the genus, subtle differences were observed in the number of intron-containing genes. These structural variations may reflect lineage-specific adaptations or underlying evolutionary differentiation among species (Liu et al. 2019; Li et al. 2024b).

Previous studies indicate that *A. muelleri*, *A. kiusianus*, *A. yunnanensis*, *A. coaetaneus*, and *A. tonkinensis* belong to the continental Asia I (CA-I) clade (Li 2024b). The close phylogenetic relationships between *A. kiusianus*, *A. yunnanensis* and *A. coaetaneus* further support their inclusion in CA-I (Gao and Yin 2024b). In the present study, *A. allenii* was confirmed to belong to the same monophyletic group in all three phylogenetic trees, indicating that these species share a recent common ancestor. Consistent with prior classifications, *A. albus*, *A. krausei*, *A. kachinensis*, and *A. konjac* are grouped together within continental Asia II (CA-II), while *A. titanum* is assigned to the SEA clade (Li 2024b). Overall, these findings are highly consistent with previous phylogenetic studies of the genus *Amorphophallus* (Pouchon et al. 2023). This framework provides a critical foundation for investigating the genus’s complex evolutionary history, including patterns of species diversification, biogeographic dispersal between continental East Asia and Southeast Asian islands, and the evolution of key morphological and ecological traits (Pouchon et al. 2023; Yin et al. 2023; Li et al. 2024b). Furthermore, this study will provides a reliable chloroplast genomic resource for the systematic and evolutionary studies of the genus *Amorphophallus*.

## Supplementary Material

Figure S3.jpg

Figure S1.jpg

Figure S2.jpg

Supplemental material.docx

## Data Availability

The chloroplast genome sequence of *Amorphophallus allenii* was deposited into the NCBI GenBank database under the accession number PX769913. The associated BioProject, Bio-Sample, and SRA numbers are PRJNA1395347, SAMN54360184, and SRR36636480, respectively.
