# Supplementary figures and images for: The complete plastome sequence of Amorphophallus allenii sheds light on intrageneric phylogeny of Amorphophallus
Source: Mitochondrial DNA B Resour. 2026 Jun 8;11(7):833–8. doi: 10.1080/23802359.2026.2680786 (PMC13248494; doi:10.1080/23802359.2026.2680786)

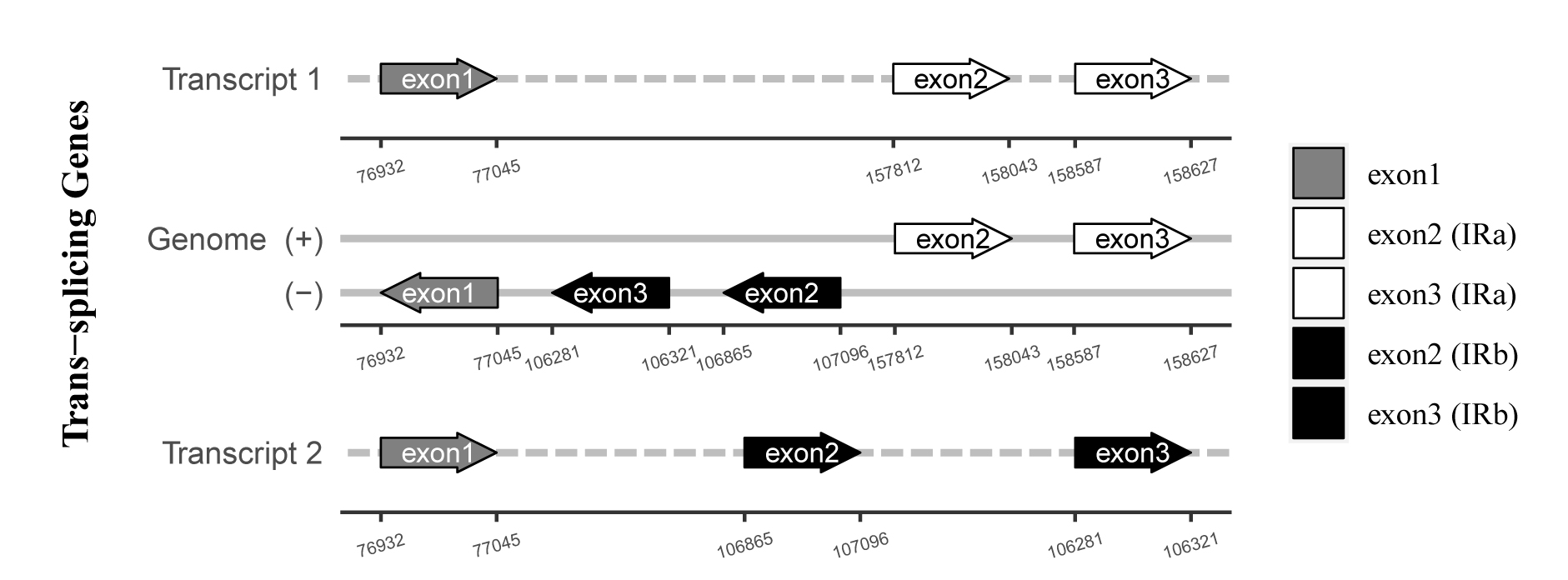

Supplement: Figure S3.jpg [file TMDN_A_2680786_SM6888.jpg]

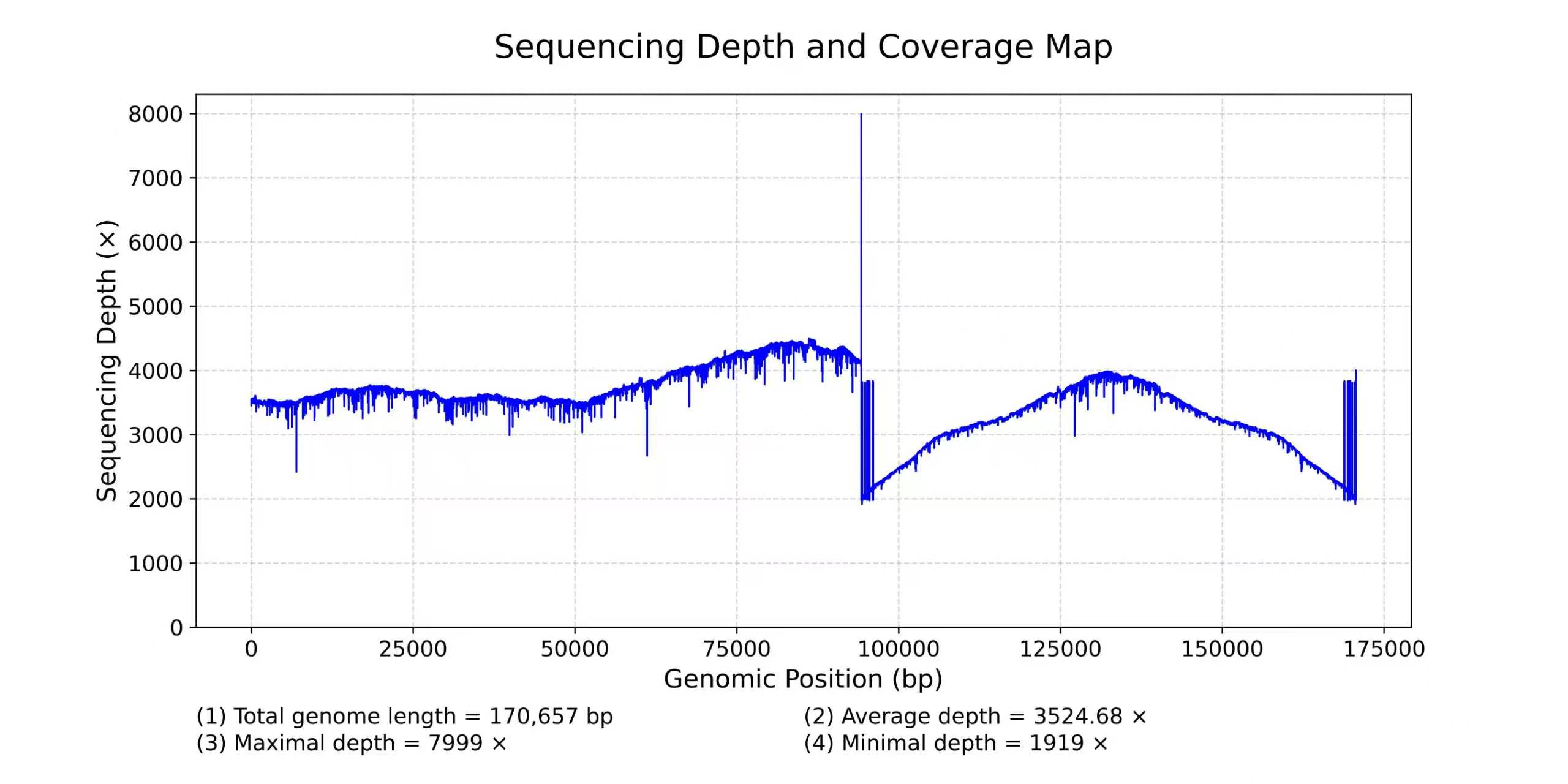

Supplement: Figure S1.jpg [file TMDN_A_2680786_SM6887.jpg]

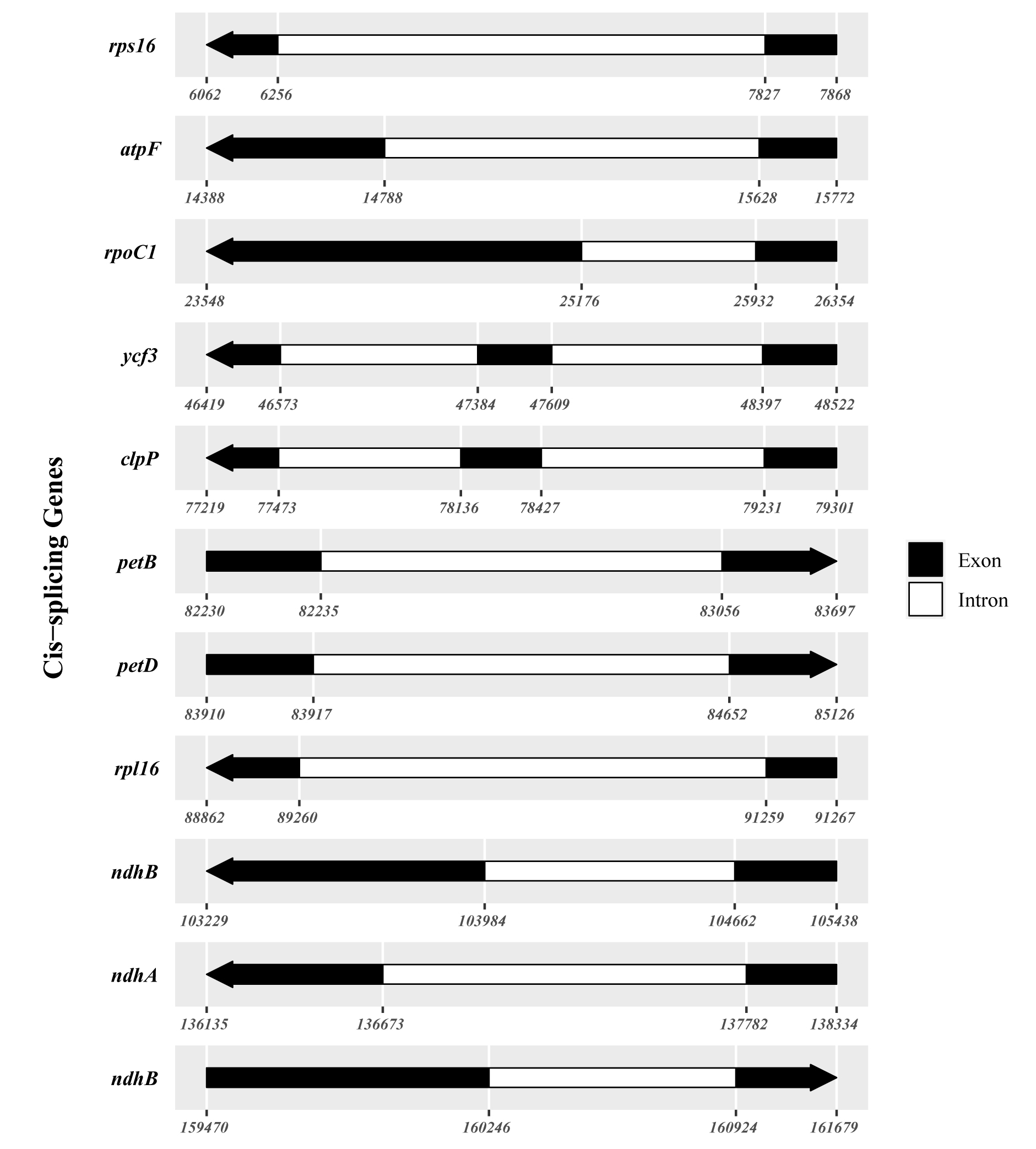

Supplement: Figure S2.jpg [file TMDN_A_2680786_SM6886.jpg]
