# Supplementary material for: The complete plastome sequence of Amorphophallus allenii sheds light on intrageneric phylogeny of Amorphophallus
Source: Mitochondrial DNA B Resour. 2026 Jun 8;11(7):833–8. doi: 10.1080/23802359.2026.2680786 (PMC13248494; doi:10.1080/23802359.2026.2680786)
Supplement: Supplemental material.docx [file TMDN_A_2680786_SM6885.docx]

Table S1 Annotated genes in chloroplast genome of *A*. *allenii.*

| Category | Gene Group | Gene Name | Number |
| --- | --- | --- | --- |
| Photosynthesis | Subunits of photosystem I | *psaB*, *psaA*, *psaI*, *psaJ*, *psaC* | 5 |
|  | Subunits of photosystem II | *psbA*, *psbK*, *psbI*, *psbM*, *psbD*, *psbC*, *psbZ*, *psbJ*, *psbL*, *psbF*, *psbE*, *psbB*, *psbT*, *psbN*, *psbH* | 15 |
|  | Subunits of NADH dehydrogenase | *ndhJ*, *ndhK*, *ndhC*, *ndhB*(2)#, *ndhF*, *ndhD*, *ndhE*, *ndhG*, *ndhI*, *ndhA*#, *ndhH* | 12 |
|  | Subunits of cytochrome b/f complex | *petN*, *petA*, *petL*, *petG*, *petB*#, *petD*# | 6 |
|  | Large subunit of rubisco | *rbcL* | 1 |
|  | Subunits of ATP synthase | *atpA*, *atpF*#, *atpH*, *atpI*, *atpE*, *atpB* | 6 |
| Self-replication | Proteins of large ribosomal subunit | *rpl33*, *rpl20*, *rpl36*, *rpl14*, *rpl16*#, *rpl22*, *rpl32* | 7 |
|  | Proteins of small ribosomal subunit | *rps12*(2)##, *rps16*#, *rps2*, *rps14*, *rps4*, *rps18*, *rps11*, *rps8*, *rps3*, *rps19*, *rps7*(2), *rps15* | 14 |
|  | Subunits of RNA polymerase | *rpoC2*, *rpoC1*#, *rpoB*, *rpoA* | 4 |
|  | Ribosomal RNAs | *rrn16*(2), *rrn23*(2), *rrn4*.*5*(2), *rrn5*(2) | 8 |
|  | Transfer RNAs | *trnH*-*GUG*, *trnK*-*UUU*#, *trnQ*-*UUG*, *trnS*-*GCU*, *trnG*-*UCC*#, *trnR*-*UCU*, *trnC*-*GCA*, *trnD*-*GUC*, *trnY*-*GUA*, *trnE*-*UUC*, *trnT*-*GGU*, *trnS*-*UGA*, *trnG*-*GCC*, *trnfM*-*CAU*, *trnM*-*CAU*(3), *trnS*-*GGA*, *trnT*-*UGU*, *trnL*-*UAA*#, *trnF*-*GAA*, *trnV*-*UAC*#, *trnW*-*CCA*, *trnP*-*UGG*, *trnL*-*CAA*(2), *trnV*-*GAC*(2), *trnI*-*GAU*(2)#, *trnA*-*UGC*(2)#, *trnR*-*ACG*(2), *trnN*-*GUU*(2), *trnL*-*UAG* | 37 |
| Other genes | Maturase | *matK* | 1 |
|  | Protease | *clpP*## | 1 |
|  | Envelope membrane protein | *cemA* | 1 |
|  | Acetyl-CoA carboxylase | *accD* | 1 |
|  | c-type cytochrome synthesis gene | *ccsA* | 1 |
|  | Conserved open reading frames | *ycf3*##, *ycf4*, *ycf2*(2), *ycf1*(2) | 6 |
| Total |  |  | 126 |

#: Intron number, (n): Gene copy number
